# Association of baseline non-severe anemia with clinical outcomes following the first recorded diagnosis of systemic sclerosis: A matched cohort study

**DOI:** 10.1097/MD.0000000000051159

**Published:** 2026-10-16

**Authors:** Yi-Chen Lai, Hsiu-Lan Weng, Kuo-Chuan Hung, I-Chia Teng, Kuo-Mao Lan, I-Wen Chen

**Affiliations:** a Department of Anesthesiology, Chi Mei Medical Center, Tainan, Taiwan; b Department of Anesthesiology, E-Da Hospital, I-Shou University, Kaohsiung, Taiwan; c School of Medicine, College of Medicine, National Sun Yat-sen University, Kaohsiung, Taiwan; d Department of Anesthesiology, Chi Mei Hospital, Liouying, Tainan, Taiwan.

**Keywords:** acute kidney injury, anemia, mortality, sepsis, systemic sclerosis

## Abstract

Systemic sclerosis (SSc) carries the highest mortality among systemic autoimmune diseases, yet the prognostic significance of anemia around the time of the first recorded SSc diagnosis is unclear. We examined whether baseline anemia is associated with early mortality and complications. This retrospective matched cohort study identified female adults with a first recorded SSc diagnosis between January 2010 and December 2024 from the TriNetX Research Network. Patients were required to have at least 5 years of pre-index observation without a documented SSc diagnosis. The network comprised 140 healthcare organizations, with approximately 70% of patients in the United States across all 9 census regions and 30% at international sites. Anemia was defined as hemoglobin (Hb) ≥8.0 and <12.0 g/dL and non-anemia as ≥12.0 g/dL, measured within ±30 days of diagnosis. For the Hb-stratified analysis, the anemia cohort was categorized as Hb ≥10.0 to <12.0 g/dL or ≥8.0 to <10.0 g/dL. One-to-one propensity score matching balanced baseline characteristics, and Cox proportional hazards models estimated hazard ratios (HRs) with 95% confidence intervals (CIs). The primary outcome was all-cause mortality during days 31 to 365 among patients surviving the 30-day landmark period; secondary outcomes were sepsis, acute kidney injury, intensive care unit admission, pulmonary fibrosis, and elevated C-reactive protein (≥10 mg/L). After matching, 5410 patients remained in each cohort. Baseline anemia was significantly associated with higher 1-year mortality (HR, 2.54; 95% CI, 1.99–3.22; *P* < .001) and with increased risks of sepsis (HR, 3.20; 95% CI, 2.38–4.30), acute kidney injury (HR, 2.24; 95% CI, 1.80–2.78), intensive care unit admission (HR, 1.92; 95% CI, 1.47–2.51), and elevated C-reactive protein (HR, 1.55; 95% CI, 1.37–1.74; all *P* < .001); no significant association was observed for pulmonary fibrosis (HR, 1.06; 95% CI, 0.96–1.17; *P* = .229). Associations persisted in sensitivity analyses and over 3-year follow-up. Mortality risk showed a graded pattern across Hb strata (Hb ≥10.0–<12.0 g/dL: HR, 1.66; Hb ≥8.0–<10.0 g/dL: HR, 3.33). Baseline anemia was associated with increased mortality and acute systemic complications following the first recorded SSc diagnosis; these observational findings warrant prospective validation.

## 1. Introduction

Systemic sclerosis (SSc) is a chronic autoimmune disease characterized by vasculopathy, immune dysregulation, and progressive fibrosis of the skin and internal organs.^[1–3]^ SSc has the highest mortality among systemic autoimmune diseases,^[4–6]^ with a 2.5- to 3-fold higher mortality rate than the general population.^[7–9]^ Traditionally, SSc-related mortality has been attributed mainly to progressive cardiopulmonary complications, including interstitial lung disease, pulmonary arterial hypertension, and cardiac involvement.^[10–12]^ However, much of this evidence derives from long-term cohorts of patients with established disease.^[8,9]^ Such designs may underestimate early mortality and obscure risk profiles at diagnosis. Emerging inception-cohort data suggest that early mortality in SSc may be driven by mechanisms different from those of long-term outcomes.^[13–15]^ Within the first year after diagnosis, patients may be vulnerable to serious infections (including sepsis) and acute renal events (e.g., scleroderma renal crisis or acute kidney injury [AKI]).^[13,15,16]^

SSc is female-predominant, with women accounting for approximately 75% to 85% of patients.^[17–19]^ Women are also at intrinsically higher risk of anemia,^[20,21]^ which may compound vulnerability in this predominantly female disease. Current evidence indicates that anemia is associated with adverse outcomes across clinical settings and may mark disease severity and physiological vulnerability, reflecting a state of low-grade systemic inflammation, impaired nutritional status, and reduced physiological reserve.^[22–24]^ In SSc, cohort studies have also shown an association between anemia and adverse outcomes.^[25–27]^ However, most evidence comes from prevalent or long-standing disease cohorts.^[25–27]^ Anemia identified around the time of the first recorded SSc diagnosis may reflect a clinically distinct state from that observed in established disease cohorts. Previous evidence from a small early SSc inception cohort identified hemoglobin (Hb) <10 g/dL as an independent predictor of mortality.^[28]^ However, its prognostic relevance in a large multinational population and its associations with acute systemic outcomes remain incompletely characterized.

We therefore hypothesized that baseline anemia is associated with increased mortality and acute complications following the first recorded SSc diagnosis. We conducted a retrospective matched cohort study using a large multicenter database to evaluate associations between anemia around the time of the first recorded diagnosis and subsequent outcomes, including all-cause mortality, sepsis, intensive care unit (ICU) admission, AKI, pulmonary fibrosis, and systemic inflammatory burden.

## 2. Methods

### 2.1. Study design and data source

This retrospective matched cohort study utilized electronic health record (EHR) data from the TriNetX Research Network, a federated database integrating deidentified clinical information from 140 healthcare organizations (HCOs) in the United States and internationally. The cohort was geographically diverse: approximately 70% of patients were contributed by US HCOs spanning all 9 census regions, and approximately 30% by international HCOs. The platform provides longitudinal data on demographics, diagnoses coded using standardized terminologies, procedures, medications, and laboratory results. Participating institutions included academic medical centers and community-based healthcare systems. TriNetX has been widely used in peer-reviewed research across medical disciplines as a real-world data source.^[29–31]^ The Chi Mei Medical Center Institutional Review Board approved the protocol and waived informed consent given the retrospective, anonymized data.

### 2.2. Inclusion criteria

Adult patients (aged ≥18 years) with a first recorded diagnosis of SSc were identified between January 1, 2010, and December 31, 2024. SSc was defined using the International Classification of Diseases, Tenth Revision, Clinical Modification code M34. The cohort was restricted to female patients given the female predominance of SSc and to reduce sex-based heterogeneity in anemia prevalence and presentation. Patients were required to have at least 5 years of pre-index observation and no documented SSc diagnosis during this period to reduce the likelihood of including prevalent SSc recorded within the available EHR history.

Baseline anemia status was determined from Hb measurements within ±30 days of the first recorded SSc diagnosis, which served as the index date. Patients were classified into the anemia cohort if their Hb concentration was ≥8.0 and <12.0 g/dL, with no concurrent Hb value ≥12.0 g/dL during the same assessment window. Patients with Hb <8.0 g/dL were excluded. Controls were required to have Hb ≥12.0 g/dL and no documented value <12.0 g/dL in the same window. This minimized exposure misclassification and ensured clear cohort separation at baseline.

### 2.3. Exclusion criteria

Exclusions were applied to reduce confounding from advanced comorbidity and acute critical illness at baseline. Patients were excluded for advanced chronic kidney disease (CKD; stage 4 or 5), end-stage renal disease, or renal replacement therapy before the index date. To avoid reverse causation, individuals with AKI, sepsis, severe sepsis, respiratory failure, critical care admission, or serum lactate ≥4.0 mmol/L within ±30 days of the index date were also excluded. In addition, patients with documented gastrointestinal bleeding in the year before or month after the index date were excluded to reduce misclassification of anemia from acute blood loss rather than disease activity. Consistent with the landmark approach, patients who died on or before day 30 were excluded, ensuring that baseline anemia status preceded outcome ascertainment and excluding early catastrophic events unlikely to be influenced by baseline hematologic status.

### 2.4. Propensity score matching

Patients were matched 1:1 by propensity score using a greedy nearest-neighbor algorithm without replacement. Propensity scores were estimated using multivariable logistic regression with exposure status as the dependent variable. The model included demographic factors (age, race/ethnicity, and body mass index [≥30 kg/m^2^]) and comorbidities (essential hypertension, dyslipidemia, neoplasms, thyroid disorders, diabetes mellitus, vitamin D deficiency, ischemic heart disease, overweight/obesity, heart failure, liver disease, CKD, cerebrovascular disease, chronic obstructive pulmonary disease [COPD], long-term systemic steroid use, nicotine dependence, COVID-19, malnutrition, respiratory failure, pulmonary embolism, and alcohol-related disorders). Laboratory measures included estimated glomerular filtration rate (≥60 mL/min/1.73 m^2^), serum albumin (≥3.5 g/dL), hemoglobin A1c (≥7%), and C-reactive protein (CRP; ≥10 mg/L). Baseline medication use included cardiovascular medications, antidepressants, angiotensin-converting enzyme inhibitors, angiotensin II receptor blockers, blood glucose-lowering drugs, insulins and analogs, antipsychotics, and biguanides. Neoplasms were not part of the exclusion criteria; their baseline presence was instead included as a model covariate to account for potential confounding. The TriNetX platform estimates this logistic regression model using the scikit-learn package in Python (Python Software Foundation), with patient record order randomized to eliminate processing-order bias. A caliper of 0.1 standard deviations of the logit of the propensity score was applied. Covariate balance was assessed by standardized mean differences (<0.1 indicating adequate balance), and propensity score distributions were examined for overlap. Detailed coding definitions are provided in Supplemental Table 1, Supplemental Digital Content 1.

### 2.5. Outcome assessment

The primary outcome was all-cause mortality during days 31 to 365 after the index date among patients surviving the 30-day landmark period. Because the primary endpoint captured death from any cause, information on the specific cause of death was not required for this analysis. Secondary outcomes were selected based on established pathophysiological links between anemia and organ dysfunction in SSc. These included ICU admission as a marker of disease severity, sepsis reflecting immune dysregulation and infection susceptibility, pulmonary fibrosis as a hallmark manifestation of disease progression, AKI representing renal involvement and systemic disease severity, and elevated CRP levels (≥10 mg/L) as an indicator of systemic inflammatory burden. Follow-up began 30 days after the index date to minimize the immortal time bias inherent to the landmark design. Patients were followed up until outcome occurrence, death, or the end of follow-up. Patients without a recorded outcome, death, or further documented healthcare encounters within the TriNetX network by the end of the follow-up window were right-censored at the date of their last observed encounter. All outcomes were also examined over 3 years to assess the persistence of associations beyond 1 year.

### 2.6. Sensitivity and hemoglobin-stratified analyses

Three sensitivity analyses were performed to assess the robustness of primary findings. Model I restricted the cohort to patients diagnosed with SSc between 2018 and 2024 to account for temporal changes in diagnosis, coding, and treatment. Model II required at least 1 confirmatory Hb measurement during months 1 to 12 of follow-up (<12 g/dL for the anemia group, ≥12 g/dL for controls) to ensure exposure consistency. Model III was limited to patients aged ≥50 years to reduce confounding from menstruation-related anemia in premenopausal women. For the Hb-stratified analysis, the anemia cohort was categorized as Hb ≥10.0 to <12.0 g/dL or ≥8.0 to <10.0 g/dL. Consistent with the primary analysis, patients with Hb <8.0 g/dL were excluded from this categorization. Because these study-defined Hb strata do not correspond directly to the 2024 World Health Organization classification of anemia severity, the strata are reported according to their measured Hb ranges rather than labeled as mild, moderate, or severe anemia.^[32]^ Each Hb stratum was separately matched 1:1 against controls, so their sample sizes do not sum to the primary matched cohort.

### 2.7. Statistical analysis

Continuous variables are presented as mean ± standard deviation, and categorical variables as frequencies (%). Missing covariate data were handled using an available-case approach consistent with the TriNetX data structure. Associations with time-to-event outcomes were estimated using Cox proportional hazards models and reported as hazard ratios (HRs) with 95% confidence intervals (CIs). The proportional hazards assumption was evaluated using the proportionality test implemented in TriNetX, with no evidence of violation for the primary 1-year outcome analyses (all *P* > .05). The TriNetX built-in Cox analysis uses an unstratified model and does not explicitly account for within-pair dependence through matched-pair stratification or cluster-robust standard errors. Significance for the primary outcome was two-sided *P* < .05; secondary-outcome analyses were exploratory and not adjusted for multiple comparisons. To assess the prognostic value of baseline anemia, a multivariable Cox model for 1-year mortality was adjusted for age, heart failure, malnutrition, neoplasms, COPD, diabetes mellitus, essential hypertension, ischemic heart disease, CKD, liver disease, overweight and obesity, long-term systemic steroid use, nicotine dependence, and available SSc-specific manifestations, including SSc with lung involvement, secondary pulmonary hypertension, esophageal dyskinesia, and cardiomyopathy. *E*-values were calculated using the online *E*-value calculator (https://www.evalue-calculator.com/evalue/) to assess robustness to unmeasured confounding. All analyses used the integrated tools of the TriNetX Research Network.

## 3. Results

### 3.1. Patient selection and baseline characteristics

From the TriNetX Research Network, we identified 5976 female adult patients with a first recorded SSc diagnosis and baseline anemia, and 12,628 without anemia who met all eligibility criteria (Fig. 1). After 1:1 propensity score matching, 5410 patients remained in each cohort. Before matching, patients with anemia were slightly older (56.7 ± 17.4 vs 54.5 ± 16.2 years) and more frequently had essential hypertension (33.8% vs 25.8%), neoplasms (21.2% vs 19.0%), heart failure (10.8% vs 5.3%), CKD (8.7% vs 3.4%), and malnutrition (4.3% vs 1.1%). Laboratory parameters also differed, with the anemia group showing lower rates of preserved renal function (74.6% vs 81.4%) and normal albumin (64.8% vs 76.0%) and a higher baseline inflammatory burden, indicated by elevated CRP (19.6% vs 11.4%; Table 1).

**Table 1:** Baseline characteristics according to anemia status at the first recorded systemic sclerosis diagnosis.

Variables	Before matching			After matching		
	Anemia group (n = 5976)	Control group (n = 12,628)	SMD	Anemia group (n = 5410)	Control group (n = 5410)	SMD
Age, yr	56.7 ± 17.4	54.5 ± 16.2	0.135	56.4 ± 17.5	56.3 ± 16.4	0.006
BMI ≥30 kg/m^2^	1316 (22.0)	2984 (23.6)	0.038	1179 (21.8)	1212 (22.4)	0.015
White	2974 (49.8)	7643 (60.5)	0.218	2815 (52.0)	2813 (52.0)	0.001
Unknown race	1085 (18.2)	2581 (20.4)	0.058	1035 (19.1)	1011 (18.7)	0.011
Black or African American	1294 (21.7)	1122 (8.9)	0.361	976 (18.0)	997 (18.4)	0.010
Asian	342 (5.7)	727 (5.8)	0.001	326 (6.0)	323 (6.0)	0.002
Comorbidities						
Essential (primary) hypertension	2019 (33.8)	3259 (25.8)	0.175	1716 (31.7)	1738 (32.1)	0.009
Dyslipidemia	1241 (20.8)	2617 (20.7)	0.001	1108 (20.5)	1071 (19.8)	0.017
Neoplasms	1265 (21.2)	2397 (19.0)	0.055	1107 (20.5)	1091 (20.2)	0.007
Disorders of thyroid gland	1150 (19.2)	2608 (20.7)	0.035	1043 (19.3)	1015 (18.8)	0.013
Diabetes mellitus	750 (12.6)	1277 (10.1)	0.077	646 (11.9)	647 (12.0)	0.001
Vitamin D deficiency	652 (10.9)	1528 (12.1)	0.037	580 (10.7)	558 (10.3)	0.013
Ischemic heart diseases	724 (12.1)	944 (7.5)	0.157	579 (10.7)	586 (10.8)	0.004
Overweight and obesity	625 (10.5)	1171 (9.3)	0.040	536 (9.9)	553 (10.2)	0.010
Heart failure	645 (10.8)	674 (5.3)	0.201	489 (9.0)	458 (8.5)	0.020
Diseases of liver	466 (7.8)	985 (7.8)	0.000	413 (7.6)	407 (7.5)	0.004
Chronic kidney disease	522 (8.7)	431 (3.4)	0.224	362 (6.7)	355 (6.6)	0.005
Cerebrovascular disease	400 (6.7)	619 (4.9)	0.077	335 (6.2)	303 (5.6)	0.026
COPD	331 (5.5)	550 (4.4)	0.055	283 (5.2)	296 (5.5)	0.011
Long-term use of systemic steroids	358 (6.0)	473 (3.7)	0.104	281 (5.2)	288 (5.3)	0.006
Nicotine dependence	304 (5.1)	691 (5.5)	0.017	267 (4.9)	275 (5.1)	0.007
COVID-19	173 (2.9)	365 (2.9)	0.000	154 (2.8)	157 (2.9)	0.003
Malnutrition	257 (4.3)	137 (1.1)	0.200	145 (2.7)	129 (2.4)	0.019
Respiratory failure	130 (2.2)	143 (1.1)	0.082	95 (1.8)	93 (1.7)	0.003
Pulmonary embolism	115 (1.9)	126 (1.0)	0.077	89 (1.6)	78 (1.4)	0.016
Alcohol-related disorders	70 (1.2)	111 (0.9)	0.029	55 (1.0)	53 (1.0)	0.004
Laboratory data						
eGFR ≥60 mL/min/1.73 m^2^	4458 (74.6)	10,283 (81.4)	0.166	4084 (75.5)	4111 (76.0)	0.012
Albumin ≥3.5 g/dL	3870 (64.8)	9591 (76.0)	0.247	3588 (66.3)	3566 (65.9)	0.009
Hemoglobin A1c ≥7%	240 (4.0)	396 (3.1)	0.047	201 (3.7)	204 (3.8)	0.003
C-reactive protein ≥10 mg/L	1174 (19.6)	1436 (11.4)	0.230	939 (17.4)	934 (17.3)	0.002
Medication						
Cardiovascular medications	3399 (56.9)	6150 (48.7)	0.164	2961 (54.7)	2942 (54.4)	0.007
Antidepressants	1413 (23.6)	2604 (20.6)	0.073	1219 (22.5)	1201 (22.2)	0.008
ACE inhibitors	678 (11.3)	939 (7.4)	0.134	552 (10.2)	566 (10.5)	0.009
Angiotensin II receptor blockers	595 (10.0)	901 (7.1)	0.101	507 (9.4)	493 (9.1)	0.009
Glucose-lowering drugs	373 (6.2)	626 (5.0)	0.056	326 (6.0)	332 (6.1)	0.005
Insulins and analogs	430 (7.2)	491 (3.9)	0.145	322 (6.0)	331 (6.1)	0.007
Antipsychotics	357 (6.0)	452 (3.6)	0.112	274 (5.1)	275 (5.1)	0.001
Biguanides	275 (4.6)	435 (3.4)	0.059	236 (4.4)	234 (4.3)	0.002

Data are presented as mean ± standard deviation or n (%). An SMD <0.1 indicates adequate covariate balance between groups.

ACE = angiotensin-converting enzyme, BMI = body mass index, COPD = chronic obstructive pulmonary disease, eGFR = estimated glomerular filtration rate, SMD = standardized mean difference.

**Figure 1.**
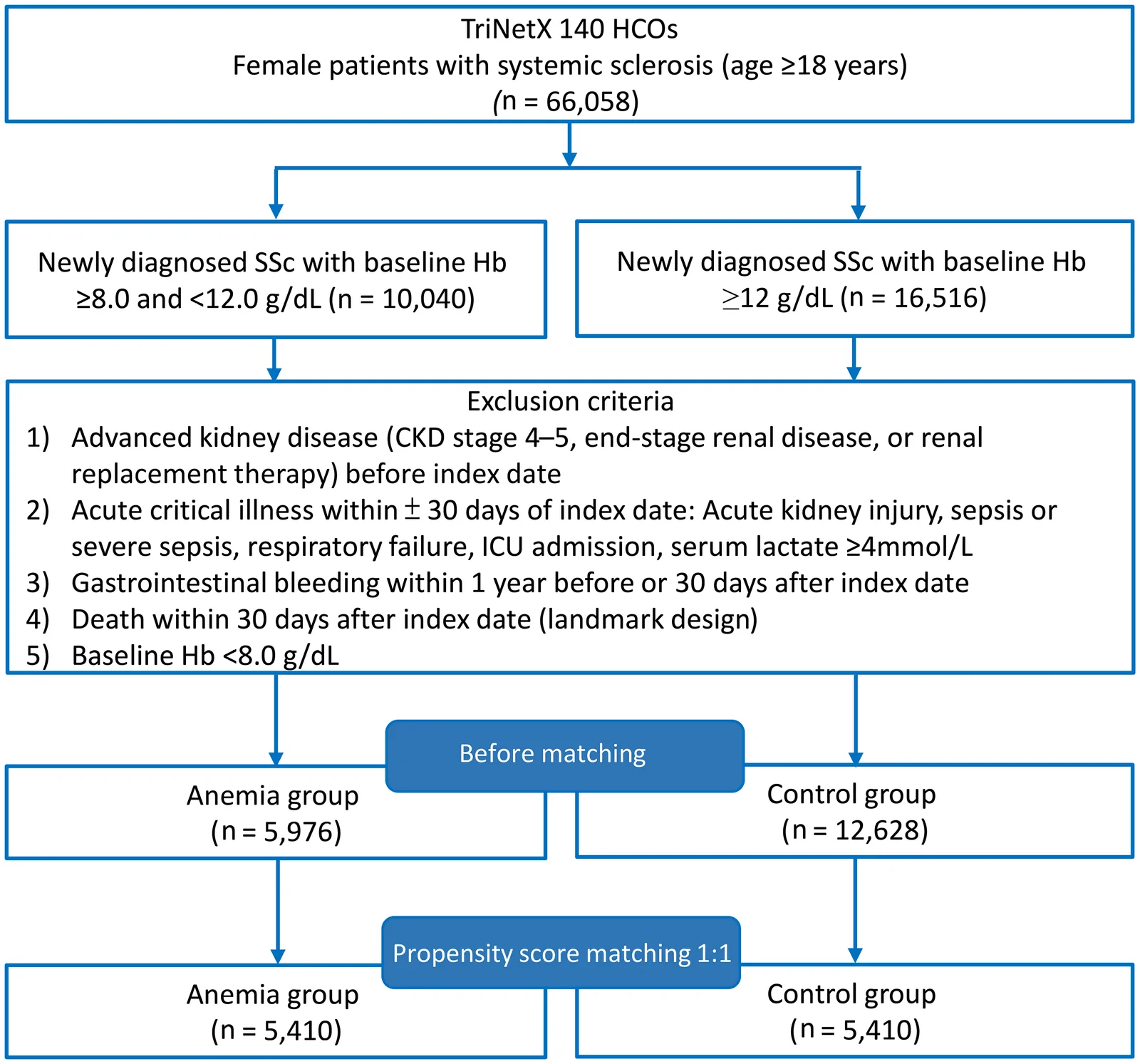
Flow diagram illustrating cohort selection from the TriNetX Research Network. CKD = chronic kidney disease, Hb = hemoglobin, HCOs = healthcare organizations, ICU = intensive care unit, SSc = systemic sclerosis.

After propensity score matching, all standardized mean differences fell below 0.1, indicating adequate covariate balance, including for neoplasms (20.5% vs 20.2%). The matched cohorts demonstrated comparable distributions of demographics, comorbidities, laboratory parameters, and medication use. Propensity score density plots confirmed substantial overlap between groups (Fig. 2).

**Figure 2.**
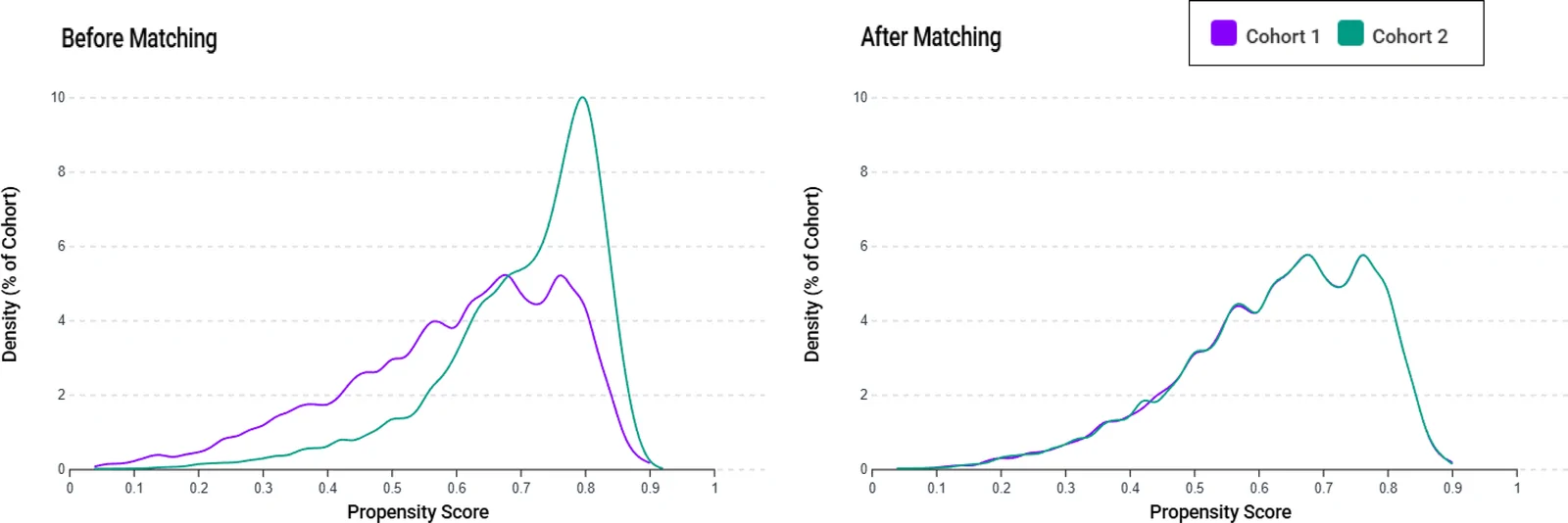
Propensity score distributions before and after matching. This figure illustrates the distribution of propensity scores for the anemia group (Cohort 1; purple line) and the control group (Cohort 2; green line) before (left panel) and after (right panel) 1:1 propensity score matching. After matching, the substantial overlap and near-superimposition of the curves indicate improved covariate balance and enhanced comparability between groups.

### 3.2. Association between anemia and 1-year outcomes

During the first year of follow-up, baseline anemia was significantly associated with elevated all-cause mortality (HR, 2.54; 95% CI, 1.99–3.22; *P* < .001; Fig. 3), with mortality more than twice that of controls (4.2% vs 1.7%; Table 2). The *E*-value was 4.52 for the point estimate and 3.39 for the lower bound of the 95% CI, indicating that an unmeasured confounder would need to be associated with both anemia and mortality by an HR of at least 4.52 to fully explain the observed association.

**Table 2:** Association between baseline anemia and clinical outcomes during days 31 to 365 after the index date among 30-day survivors (n = 5410 for each group).

Outcomes	Anemia group Events (%)	Control group Events (%)	HR (95% CI)	*P* value	*P* value for PH assumption
Mortality	227 (4.2)	94 (1.7)	2.54 (1.99–3.22)	<.001	.884
ICU admission	150 (2.8)	82 (1.5)	1.92 (1.47–2.51)	<.001	.095
Sepsis	176 (3.3)	58 (1.1)	3.20 (2.38–4.30)	<.001	.625
Pulmonary fibrosis	809 (15.0)	792 (14.6)	1.06 (0.96–1.17)	.229	.836
Acute kidney injury	252 (4.7)	119 (2.2)	2.24 (1.80–2.78)	<.001	.277
Elevated CRP (≥10 mg/L)	682 (12.6)	471 (8.7)	1.55 (1.37–1.74)	<.001	.314

CI = confidence interval, CRP = C-reactive protein, HR = hazard ratio, ICU = intensive care unit, PH = proportional hazards.

**Figure 3.**
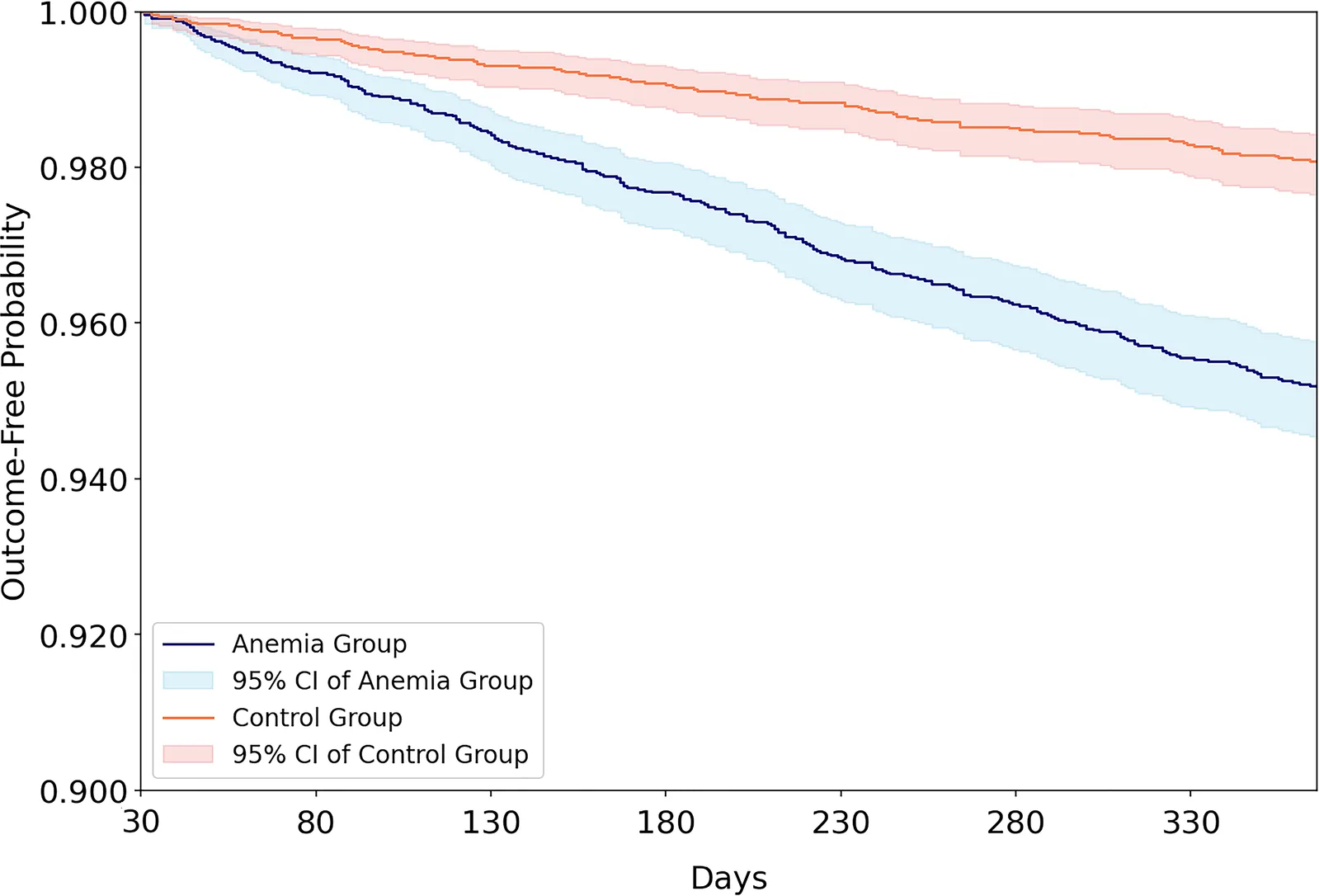
Outcome-free survival curves by anemia status. This figure depicts outcome-free survival curves for patients with anemia (anemia group; blue line) and those with normal hemoglobin levels (control group; orange line), with follow-up beginning on day 31 after the index date; the primary analysis covered days 31 to 365 among patients who survived the 30-day landmark period. Shaded areas represent 95% CIs. Over the follow-up period, patients in the anemia group consistently exhibited a lower probability of remaining outcome-free compared with controls, indicating an association between anemia and an increased risk of mortality. CI = confidence interval.

Among secondary outcomes, the strongest association emerged for sepsis (HR, 3.20; 95% CI, 2.38–4.30; *P* < .001), followed by AKI (HR, 2.24; 95% CI, 1.80–2.78; *P* < .001) and ICU admission (HR, 1.92; 95% CI, 1.47–2.51; *P* < .001). Elevated CRP levels occurred more frequently among patients with anemia (HR, 1.55; 95% CI, 1.37–1.74; *P* < .001). In contrast, pulmonary fibrosis development did not differ significantly between cohorts (HR, 1.06; 95% CI, 0.96–1.17; *P* = .229).

### 3.3. Association between anemia and extended outcomes

Over the 3-year follow-up, associations between anemia and adverse outcomes persisted with attenuated effect sizes (Table 3). Mortality remained higher in the anemia group (HR, 2.03; 95% CI, 1.75–2.36; *P* < .001; 9.2% vs 4.9%), as were sepsis (HR, 2.11; 95% CI, 1.75–2.53), AKI (HR, 2.06; 95% CI, 1.78–2.39), ICU admission (HR, 1.76; 95% CI, 1.46–2.11), and elevated CRP levels (HR, 1.43; 95% CI, 1.31–1.57; all *P* < .001). Pulmonary fibrosis remained comparable between groups (HR, 1.04; 95% CI, 0.95–1.13; *P* = .420).

**Table 3:** Association between baseline anemia and clinical outcomes during 3-year follow-up beginning on day 31 after the index date.

Outcomes	Anemia group Events (%)	Control group Events (%)	HR (95% CI)	*P* value
Mortality	500 (9.2)	267 (4.9)	2.03 (1.75–2.36)	<.001
ICU admission	300 (5.5)	185 (3.4)	1.76 (1.46–2.11)	<.001
Sepsis	334 (6.2)	173 (3.2)	2.11 (1.75–2.53)	<.001
Pulmonary fibrosis	1058 (19.6)	1079 (19.9)	1.04 (0.95–1.13)	.420
Acute kidney injury	502 (9.3)	267 (4.9)	2.06 (1.78–2.39)	<.001
Elevated CRP (≥10 mg/L)	1081 (20.0)	831 (15.4)	1.43 (1.31–1.57)	<.001

CI = confidence interval, CRP = C-reactive protein, HR = hazard ratio, ICU = intensive care unit.

### 3.4. Sensitivity analyses

The primary findings were robust across all sensitivity analyses (Table 4). The mortality association remained significant when restricting to patients diagnosed between 2018 and 2024 (Model I: HR, 2.86; 95% CI, 2.19–3.75; *P* < .001), when requiring confirmatory Hb measurements during follow-up (Model II: HR, 2.79; 95% CI, 2.08–3.73; *P* < .001), and when limiting to patients aged ≥50 years (Model III: HR, 2.30; 95% CI, 1.81–2.94; *P* < .001). Associations with sepsis, ICU admission, AKI, and elevated CRP levels remained significant across all models.

**Table 4:** Sensitivity analyses for the association between baseline anemia and clinical outcomes during days 31 to 365 among 30-day survivors.

Outcomes	Model I			Model II		Model III	
	HR (95% CI)	*P* value	HR (95% CI)		*P* value	HR (95% CI)	*P* value
Mortality	2.86 (2.19–3.75)	<.001	2.79 (2.08–3.73)		<.001	2.30 (1.81–2.94)	<.001
ICU admission	1.88 (1.43–2.48)	<.001	2.25 (1.68–3.01)		<.001	1.62 (1.21–2.16)	.001
Sepsis	2.53 (1.87–3.41)	<.001	3.50 (2.56–4.76)		<.001	2.89 (2.13–3.92)	<.001
Pulmonary fibrosis	1.05 (0.95–1.16)	.336	1.14 (1.03–1.28)		.016	1.06 (0.95–1.18)	.280
Acute kidney injury	2.47 (1.97–3.09)	<.001	2.68 (2.12–3.40)		<.001	1.96 (1.56–2.47)	<.001
Elevated CRP (≥10 mg/L)	1.64 (1.44–1.86)	<.001	1.75 (1.54–1.98)		<.001	1.62 (1.41–1.85)	<.001

Model I: restricted to patients diagnosed between 2018 and 2024 (n = 4319 per group). Model II: restricted to patients with confirmatory hemoglobin measurements during months 1 to 12 of follow-up (n = 3111 per group). Model III: restricted to patients aged ≥50 years (n = 4191 per group).

CI = confidence interval, CRP = C-reactive protein, HR = hazard ratio, ICU = intensive care unit.

### 3.5. Hemoglobin-stratified analysis

In the Hb-stratified analysis, progressively stronger associations were observed across lower Hb ranges (Table 5). Compared with non-anemic controls, mortality risk was higher among patients with Hb ≥10.0 to <12.0 g/dL (HR, 1.66; 95% CI, 1.24–2.23; *P* = .001) and was further increased among those with Hb ≥8.0 to <10.0 g/dL (HR, 3.33; 95% CI, 2.08–5.33; *P* < .001). A similar stepwise pattern was noted for sepsis (Hb ≥8.0–<10.0 g/dL: HR, 3.69; 95% CI, 2.11–6.45; *P* < .001; Hb ≥10.0–<12.0 g/dL: HR, 1.76; 95% CI, 1.24–2.49; *P* = .001). Consistent graded patterns were evident for AKI, ICU admission, and elevated CRP levels.

**Table 5:** Hemoglobin-stratified associations between baseline anemia and clinical outcomes during days 31 to 365 among 30-day survivors.

Outcomes	Hb ≥10.0–<12.0 g/dL vs control*		Hb ≥8.0–<10.0 g/dL vs control†	
	HR (95% CI)	*P* value	HR (95% CI)	*P* value
Mortality	1.66 (1.24–2.23)	.001	3.33 (2.08–5.33)	<.001
ICU admission	1.50 (1.07–2.10)	.019	2.04 (1.23–3.38)	.005
Sepsis	1.76 (1.24–2.49)	.001	3.69 (2.11–6.45)	<.001
Pulmonary fibrosis	1.11 (1.00–1.24)	.059	0.94 (0.75–1.17)	.571
Acute kidney injury	1.69 (1.31–2.20)	<.001	2.77 (1.77–4.33)	<.001
Elevated CRP (≥10 mg/L)	1.36 (1.18–1.56)	<.001	1.64 (1.27–2.13)	<.001

CI = confidence interval, CRP = C-reactive protein, Hb = hemoglobin, HR = hazard ratio, ICU = intensive care unit.

* n = 3944 per group.

† n = 1031 per group.

### 3.6. Multivariable Cox proportional hazards model

In the multivariable Cox model, including available SSc-specific manifestations, baseline anemia remained associated with 1-year mortality (HR, 2.69; 95% CI, 2.24–3.24; *P* < .001; Table 6). Other predictors of mortality were advanced age (HR, 1.03 per year; 95% CI, 1.02–1.04; *P* < .001), heart failure (HR, 1.97; 95% CI, 1.54–2.53; *P* < .001), malnutrition (HR, 2.75; 95% CI, 1.98–3.82; *P* < .001), neoplasms (HR, 1.33; 95% CI, 1.09–1.63; *P* = .005), COPD (HR, 1.38; 95% CI, 1.02–1.85; *P* = .036), and secondary pulmonary hypertension (HR, 1.56; 95% CI, 1.24–1.97; *P* < .001), whereas ischemic heart disease showed an inverse association (HR, 0.74; 95% CI, 0.56–0.97; *P* = .030). The remaining covariates, including lung involvement, esophageal dyskinesia, and cardiomyopathy, were not significantly associated with mortality.

**Table 6:** Multivariable Cox proportional hazards model for mortality during days 31 to 365 among 30-day survivors, including available SSc-specific manifestations.

Variable	HR (95% CI)	*P* value
Anemia group vs control group	2.69 (2.24–3.24)	<.001
Age at index (per year)	1.03 (1.02–1.04)	<.001
Essential hypertension	0.90 (0.73–1.10)	.283
Neoplasms	1.33 (1.09–1.63)	.005
Diabetes mellitus	0.79 (0.60–1.05)	.101
Ischemic heart disease	0.74 (0.56–0.97)	.030
Overweight and obesity	0.72 (0.52–1.01)	.060
Heart failure	1.97 (1.54–2.53)	<.001
Diseases of liver	1.08 (0.80–1.45)	.614
Chronic kidney disease	1.12 (0.83–1.50)	.466
Chronic obstructive pulmonary disease	1.38 (1.02–1.85)	.036
Long-term systemic steroid use	1.11 (0.77–1.60)	.588
Nicotine dependence	1.16 (0.80–1.68)	.434
Malnutrition	2.75 (1.98–3.82)	<.001
Systemic sclerosis with lung involvement	1.33 (0.85–2.07)	.208
Secondary pulmonary hypertension	1.56 (1.24–1.97)	<.001
Dyskinesia of esophagus	0.64 (0.36–1.12)	.116
Cardiomyopathy	1.35 (0.86–2.12)	.186

The multivariable Cox model was fitted in the unmatched cohort (source cohort, n = 18,604). The built-in TriNetX multivariable Cox output does not provide the model-specific analytic sample size or number of mortality events.

CI = confidence interval, HR = hazard ratio, SSc = systemic sclerosis.

## 4. Discussion

Whether anemia identified at the time of the first recorded SSc diagnosis remains associated with early clinical outcomes after adjustment for measured covariates has been unclear, as prior evidence derives primarily from established or long-standing disease cohorts. This multicenter matched cohort study demonstrated that baseline anemia is associated with substantially elevated risks of adverse clinical outcomes following the first recorded SSc diagnosis. The anemia group exhibited a more than 2-fold higher mortality risk during days 31 to 365 among 30-day survivors, with particularly pronounced associations observed for sepsis and AKI. Importantly, these associations persisted across extended follow-up and multiple sensitivity analyses, with a graded pattern of stronger associations across lower Hb strata. Notably, anemia showed no significant association with pulmonary fibrosis, suggesting that it may be a more specific marker for acute systemic complications than for progressive fibrotic manifestations.

SSc is a complex autoimmune disease characterized by immune dysregulation, progressive fibrosis, and widespread vasculopathy, and is associated with the highest mortality among systemic autoimmune disorders. The disease occurs more frequently in women than in men, highlighting an important sex-based disparity in the disease burden. Similarly, anemia represents a major public health concern and has been consistently associated with increased susceptibility to infection, diminished physiological reserves, and excess mortality across diverse clinical settings. Because both SSc and anemia are more prevalent in female populations and share overlapping pathophysiological consequences, including immune dysregulation, chronic inflammatory stress, and heightened vulnerability to systemic complications, investigating their relationship around the time of the first recorded SSc diagnosis may yield clinically relevant insights. Such an approach may help refine early risk stratification and inform timely interventions in the disease course.

Our observation that baseline anemia is associated with substantially increased mortality following the first recorded SSc diagnosis extends prior evidence on its prognostic relevance in SSc. An early Thai inception cohort identified Hb <10 g/dL as an independent predictor of mortality, although that study included only 115 patients.^[28]^ A French cohort study reported that SSc patients with anemia had more severe disease and that anemia at inclusion was associated with elevated mortality after multivariable adjustment.^[25]^ A Chinese retrospective study demonstrated that baseline anemia was independently related to organ damage progression in patients with SSc, with the anemia index identified as an indicator of worse organ damage trajectories.^[27]^ The present study extends these observations in a substantially larger multinational EHR-based population and evaluates several acute systemic outcomes in addition to mortality. Importantly, this mortality association was supported by consistent findings across all sensitivity analyses and by *E*-value calculations indicating that substantial unmeasured confounding would be required to fully explain it.

The pronounced association between baseline anemia and sepsis risk in our cohort deserves careful interpretation. Patients with SSc face elevated infection susceptibility through multiple disease-related mechanisms, including structural lung damage predisposing them to respiratory infections, digital ulcers serving as portals for skin and soft tissue infections, and gastrointestinal dysmotility contributing to aspiration risk and bacterial overgrowth.^[33,34]^ Additionally, immunosuppressive therapies commonly used in SSc management may further compromise the host defense. Anemia in this context may serve as an integrative marker reflecting nutritional compromise, chronic inflammation, and reduced physiological reserve, factors that collectively diminish infection resistance.

The association between baseline anemia and AKI is also biologically plausible. Renal involvement in SSc spans a continuum, ranging from overt scleroderma renal crisis to more subtle forms of microvascular dysfunction.^[35]^ Prior case–control data indicate that advanced age, preexisting CKD, and heart failure are significant risk factors for AKI in patients with SSc, with prerenal mechanisms accounting for a substantial proportion of events.^[36]^ In this context, anemia may further heighten renal vulnerability by limiting oxygen delivery to the renal parenchyma and increasing susceptibility to ischemic injury during hemodynamic stress. The interplay between SSc-related vasculopathy and anemia-associated tissue hypoxia may therefore create a high-risk milieu for acute renal complications.

The absence of a significant association between baseline anemia and pulmonary fibrosis development warrants further interpretation. Pulmonary fibrosis in SSc develops through complex pathophysiological mechanisms involving autoimmune-mediated inflammation, vascular injury, and fibroblast activation over extended periods.^[37]^ The lack of association with baseline anemia may reflect the distinct biological pathways driving pulmonary fibrosis compared to acute complications, such as sepsis or renal injury. The higher rates of elevated CRP during follow-up among anemic patients support the concept of anemia as a marker of heightened systemic inflammatory burden. Prior research showed that patients with SSc and baseline anemia more frequently had elevated inflammatory markers, including erythrocyte sedimentation rate and CRP,^[27]^ indicating that anemia was more common in patients with active disease. This inflammatory association may partially explain the observed relationships between anemia and adverse outcomes, as chronic inflammation contributes to both anemia pathogenesis and susceptibility to complications, such as infection and organ dysfunction. The persistence of this inflammatory association across follow-up periods suggests that baseline anemia may identify patients with ongoing heightened disease activity.

The graded pattern of progressively higher mortality and complication risks across lower Hb strata strengthens the plausibility of a meaningful relationship between anemia and adverse outcomes. This graded pattern has important clinical implications, suggesting that the Hb level may serve as a readily accessible and quantitative tool for risk stratification around the time of the first recorded diagnosis. Baseline anemia may help identify patients who warrant closer clinical assessment and risk evaluation, pending prospective validation of whether such approaches translate into improved outcomes. This graded pattern across Hb strata also supports including Hb assessment in the baseline evaluation following a first recorded SSc diagnosis.

Several limitations warrant acknowledgment. First, as this was an observational study based on EHR data, residual confounding from unmeasured or incompletely captured variables could not be excluded. Although *E*-value analyses were performed, they could only partially address the potential impact of unmeasured confounders. Second, the retrospective design and reliance on administrative and diagnostic codes may have introduced misclassification of exposures, outcomes, and comorbidities. Although a 5-year pre-index period without a recorded SSc diagnosis was required, the first recorded M34 code may not represent true disease onset because earlier diagnoses outside participating HCOs may not have been captured. In addition, the predominantly US-based composition of the TriNetX population may limit the generalizability of our findings to other healthcare systems and ethnically or geographically distinct populations. Third, although available SSc-specific manifestations, including SSc with lung involvement, secondary pulmonary hypertension, esophageal dyskinesia, and cardiomyopathy, were incorporated into the revised multivariable model, the TriNetX database lacks sufficiently detailed disease-specific information, including modified Rodnan skin score, pulmonary function measures such as forced vital capacity and diffusing capacity of the lungs for carbon monoxide, autoantibody profiles, scleroderma renal crisis, and reliably classified limited versus diffuse cutaneous SSc. Residual confounding by disease phenotype and severity, therefore, cannot be excluded. Moreover, the etiology of anemia could not be distinguished; different subtypes, such as iron deficiency anemia, anemia of chronic disease, and microangiopathic hemolytic anemia, may reflect distinct pathophysiological processes with heterogeneous prognostic implications. Fourth, laboratory measurements and clinical events occurring outside participating HCOs may not be fully captured, potentially resulting in incomplete ascertainment of exposure and outcomes. Patients who discontinued care within the network before an outcome was recorded were censored at their last observed encounter, which may introduce informative censoring if disenrollment is related to underlying health status. Fifth, the exclusion of patients with Hb <8.0 g/dL limits the generalizability of our findings to that group. In addition, the exclusion of recent gastrointestinal bleeding and acute critical illness, together with the 30-day landmark requirement, means that the findings are most applicable to selected clinically stable 30-day survivors. These exclusions may also have omitted clinically important SSc phenotypes associated with severe anemia or gastrointestinal vascular involvement, further limiting generalizability to patients with more severe disease. Sixth, because the cohort was restricted to female patients, our findings may not generalize to male patients with SSc, who represent a smaller but clinically important subgroup with a potentially different disease course. Finally, information on disease-modifying therapies, immunosuppressive treatments, and their timing relative to diagnosis was not comprehensively available, precluding assessment of their potential modifying effects.

## 5. Conclusion

Among selected 30-day survivors following the first recorded diagnosis of SSc, non-severe baseline anemia was associated with higher risks of mortality during days 31 to 365, sepsis, AKI, and systemic inflammation. These findings are observational and do not imply causality; rather, they should be viewed as hypothesis-generating. Confirmation in prospective cohorts with detailed characterization of anemia etiology and disease phenotypes is warranted. Future investigations should also examine whether the correction of anemia modifies clinical outcomes and elucidates the biological mechanisms underlying these associations. If validated, routine Hb assessment around the time of the first recorded SSc diagnosis may aid subsequent risk stratification.

## Author contributions

**Conceptualization:** Yi-Chen Lai, Hsiu-Lan Weng, Kuo-Chuan Hung, I-Chia Teng, I-Wen Chen.

**Data curation:** Yi-Chen Lai, Hsiu-Lan Weng, Kuo-Chuan Hung, I-Chia Teng, Kuo-Mao Lan, I-Wen Chen.

**Formal analysis:** Yi-Chen Lai, Hsiu-Lan Weng, Kuo-Chuan Hung, I-Chia Teng, I-Wen Chen.

**Investigation:** Hsiu-Lan Weng, Kuo-Mao Lan.

**Methodology:** Kuo-Mao Lan.

**Resources:** Yi-Chen Lai, I-Wen Chen.

**Software:** Yi-Chen Lai, I-Wen Chen.

**Validation:** I-Chia Teng, Kuo-Mao Lan.

**Writing – original draft:** Yi-Chen Lai, Kuo-Chuan Hung, I-Wen Chen.

**Writing – review & editing:** Yi-Chen Lai, Kuo-Chuan Hung, I-Wen Chen.


