# Supplementary material for: Association of baseline non-severe anemia with clinical outcomes following the first recorded diagnosis of systemic sclerosis: A matched cohort study
Source: Medicine (Baltimore). 2026 Oct 16;105(42):e51159. doi: 10.1097/MD.0000000000051159 (PMC13681093; doi:10.1097/MD.0000000000051159)
Supplement: Supplementary file 1 [file medi-105-e51159-s001.docx]

**Supplemental Table 1. Coding Definitions for Cohort Construction, Outcomes, and Propensity Score Matching**

| Category | Variable / Condition | Coding Definition |
| --- | --- | --- |
| Cohort Identification | Systemic sclerosis (SSc) | ICD-10-CM: M34 |
|  | Age | ≥18 years at index date |
|  | Sex | Female |
| Index Date Definition | Newly diagnosed SSc | First recorded ICD-10-CM M34 during study period (2010–2024), with no prior SSc diagnosis |
| Exposure Definition | Baseline anemia | Hemoglobin ≥8.0 and <12.0 g/dL within ±30 days of index date; no hemoglobin ≥12 g/dL in same window |
| Control Definition | Non-anemia | Hemoglobin ≥12 g/dL within ±30 days of index date; no hemoglobin <12.0 g/dL in same window |
| Key Exclusion Criteria | Advanced chronic kidney disease | ICD-10-CM: N18.4 (CKD stage 4), N18.5 (CKD stage 5), N18.6 (ESRD), Z99.2 (dialysis dependence) |
|  | Acute kidney injury | ICD-10-CM: N17 |
|  | Sepsis / severe sepsis | ICD-10-CM: A41, R65.2 |
|  | Respiratory failure | ICD-10-CM: J96 |
|  | Critical illness | CPT: 1013729 (Critical care services) |
|  | Hyperlactatemia | Lactate ≥4.0 mmol/L |
|  | Early mortality | Death within 30 days after index date |
|  | Gastrointestinal bleeding | ICD-10-CM: K92.0, K92.1, K92.2, K31.81 |
| Outcomes | All-cause mortality | Demographic death indicator; ICD-10-CM: R99 |
|  | ICU admission | CPT: 1013729 |
|  | Sepsis | ICD-10-CM: A41, R65.2 |
|  | Acute kidney injury | ICD-10-CM: N17 |
|  | Pulmonary fibrosis | ICD-10-CM: J84.1, J84.8, J84.9 |
|  | Inflammatory activity | C-reactive protein ≥10 mg/L |
| Propensity Score Matching Variables | Demographics | Age, race/ethnicity, body mass index |
|  | Cardiovascular comorbidities | Hypertension (I10), ischemic heart disease (I20–I25), heart failure (I50) |
|  | Metabolic conditions | Diabetes mellitus (E08–E13), dyslipidemia (E78), obesity (E66), disorders of thyroid gland (E00–E07), vitamin D deficiency (E55.9) |
|  | Renal disease | Chronic kidney disease (N18) |
|  | Pulmonary disease | Chronic obstructive pulmonary disease (J44), respiratory failure (J96), pulmonary embolism (I26) |
|  | Other comorbidities | Malignancy (C00–D49), liver disease (K70–K77), malnutrition (E40–E46), cerebrovascular disease (I60–I69), COVID-19 (U07.1), alcohol-related disorders (F10) |
|  | Medications | Cardiovascular medications, ACE inhibitors, angiotensin II receptor blockers, antidepressants, antipsychotics, antidiabetic agents |
|  | Laboratory parameters | Estimated glomerular filtration rate, serum albumin, C-reactive protein, hemoglobin A1c (≥7%) |

CKD, chronic kidney disease; CPT, Current Procedural Terminology; ESRD, end-stage renal disease; ICD-10-CM, International Classification of Diseases, Tenth Revision, Clinical Modification; ICU, intensive care unit; SSc, systemic sclerosis.
